# The Pathological Anatomy of the Human Body

**Published:** 1847-01

**Authors:** 


Art. XV.—*The Pathological Anatomy of the Human Body.* By Julius Vogel, M.D. Translated by G. E. Day, M.A. and L.M. Cantab. Illustrated by Ten Plates.—*London,* 1847. 8vo, pp. 587.

In our last Number we gave a pretty full analysis of the original of this very valuable work, to which we must refer the reader. We have only to add here our opinion that the translator has performed his task in an excellent manner, and has enriched the work with many valuable additions.

